# FAM49B promotes breast cancer proliferation, metastasis, and chemoresistance by stabilizing ELAVL1 protein and regulating downstream Rab10/TLR4 pathway

**DOI:** 10.1186/s12935-021-02244-9

**Published:** 2021-10-13

**Authors:** Yanhui Li, Yue Xiong, Zhen Wang, Jianjun Han, Sufang Shi, Jinglan He, Na Shen, Wenjuan Wu, Rui Wang, Weiwei Lv, Yajun Deng, Weiguang Liu

**Affiliations:** 1 Clinical School of Medicine, Hebei University of Engineering, Handan, 056000 Hebei China; 2 Department of Laboratory Medicine, Medical College, Hebei University of Engineering, Handan, 056000 Hebei China; 3 Department of Breast Surgery, Affiliated Hospital of Hebei University of Engineering, Handan, 056000 Hebei China; 4 Department of Orthopedic Surgery, Affiliated Hospital of Hebei University of Engineering, Handan, 056000 Hebei China; 5 Science and Education Division, Affiliated Hospital of Hebei University of Engineering, Handan, 056000 Hebei China

**Keywords:** Breast cancer, FAM49B, ELAVL1, Rab10, TLR4, Proliferation, Metastasis, Chemoresistance

## Abstract

**Background:**

Breast cancer (BC) is one of the most common cancers and the leading cause of death in women. Previous studies have demonstrated that FAM49B is implicated in several tumor progression, however, the role and mechanism of FAM49B in BC remain to be explored. Therefore, in this study, we aimed to systematically study the role of FAM49B in the proliferation, metastasis, apoptosis, and chemoresistance of BC, as well as the corresponding molecular mechanisms and downstream target.

**Methods:**

The ONCOMINE databases and Kaplan–Meier plotter databases were analyzed to find FAM49B and its prognostic values in BC. FAM49B expression in BC and adjacent non-tumor tissues was detected by western blot and IHC. Kaplan–Meier analysis was used to identify the prognosis of BC patients. After FAM49B knockdown in MCF-7 and MDA-MB-231 cells, a combination of co-immunoprecipitation, MTT, migration, and apoptosis assays, nude mouse xenograft tumor model, in addition to microarray detection and data analysis was used for further mechanistic studies.

**Results:**

In BC, the results showed that the expression level of FAM49B was significantly higher than that in normal breast tissue, and highly expression of FAM49B was significantly positively correlated with tumor volume, histological grade, lymph node metastasis rate, and poor prognosis. Knockdown of FAM49B inhibited the proliferation and migration of BC cells in vitro and in vivo. Microarray analysis revealed that the Toll-like receptor signaling pathway was inhibited upon FAM49B knockdown. In addition, the gene interaction network and downstream protein validation of FAM49B revealed that FAM49B positively regulates BC cell proliferation and migration by promoting the Rab10/TLR4 pathway. Furthermore, endogenous FAM49B interacted with ELAVL1 and positively regulated Rab10 and TLR4 expression by stabilizing ELAVL1. Moreover, mechanistic studies indicated that the lack of FAM49B expression in BC cells conferred more sensitivity to anthracycline and increased cell apoptosis by downregulating the ELAVL1/Rab10/TLR4/NF-κB signaling pathway.

**Conclusion:**

These results demonstrate that FAM49B functions as an oncogene in BC progression, and may provide a promising target for clinical diagnosis and therapy of BC.

## Background

Breast cancer (BC) is currently the most common cancer among women female, with approximately 684,996 522,000 deaths worldwide in 2020 [1]. Just like many cancers, breast cancer is affected by many endocrine disruptors during its occurrence. Lifestyle factors will aggravate human exposure to chemicals. Therefore, breast cancer is a disease caused by multiple factors [2]. Comprehensive treatments such as chemotherapy, targeted therapy, biological therapy and immunotherapy can significantly improve the prognosis of breast cancer patients [3]. Traditional chemotherapy has been developed in a more efficient and precise direction [4, 5]. The development of the disease and treatment monitoring tools, however, are slowed down, and due to the biological complexity of BC, it is difficult to fully evaluate the treatment response, metastasis pattern, and clinical outcomes using the most common clinicopathological markers [estrogen receptor (ER), progesterone receptor (PR), human epidermal growth factor receptor 2 (HER2), and Ki-67] [6]. Therefore, identifying new and effective biomarkers could improve BC clinical behavior and provide new therapeutic strategies.

The family with sequence similarity 49 member B (FAM49B) gene is a member of FAM49 in vertebrates. FAM49A and FAM49C are the other two members of the FAM49 gene family. They all have a conservative domain of DUF 1394. FAM49A and FAM49B only exist in the human genome, whereas FAM49C only exists in fishes, amphibians, reptiles, birds, and some lower mammals [7]. Currently, there are few reported studies on the FAM49B gene in tumors. Some studies suggest that FAM49B is a regulator of actin kinetics, and T cell activation is related [8], which participates in mitochondrial function and inhibits proliferation and metastasis of pancreatic cancer [9]. In addition, FAM49B is a downstream target of ZFR and a potential tumor suppressor in colorectal and liver cancers [10]. However, a recent study revealed that FAM49B promotes gallbladder carcinoma cell proliferation and migration. Inhibition of PI3K/AKT pathway via FAM49B expression abrogated Myc-TASP1/Lv-shTASP1-induced gallbladder carcinoma cell proliferation and motility [11]. The above studies suggest that FAM49B may have various important tissue-specific functions, and its deregulation could promote or inhibit the emergence and evolution of some diseases, including cancer. However, the mechanism by which FAM49B plays its role in the genesis, development, and outcome of BC remains unclear. In our previous work, we found that TUFT1 is an oncogene in BC, we analyzed the downstream high-throughput data of TUFT1 and found that FAM49B mRNA is also significantly increased in BC, suggesting that FAM49B mRNA may also contribute to the occurrence and development of BC. Not a small impact [12]. We analyzed differentially expressed in cancer and adjacent tissues using the ONCOMINE database, which contains data from clinical BC samples. This indicated that the expression of FAM49B was significantly higher than that in normal breast tissue in many BC samples. Therefore, in this study, we aimed to further analyze the expression pattern of FAM49B in BC, investigate its biological functions, and correlate these findings with BC patient prognosis.

## Methods

### Human specimens

We enrolled 180 patients with invasive BC who underwent a breast surgery at the Affiliated Hospital of the Hebei University of Engineering between January 2012 and December 2014; moreover, eight more patients were enrolled between January and February 2018. The selection criteria were as described previously [12, 13]. Written informed consent was obtained from all participants, based on the Declaration of Helsinki. The protocol was approved by the Ethics Committee of the Affiliated Hospital of Hebei University of Engineering.

### Human BC cell lines

MCF-7, MDA-MB-231, MDA-MB-361, HCC1937, and SKBR-3 cell lines were obtained from the American Type Culture Collection (USA). The cancer cell lines were cultured in RPMI-1640 supplemented with 10% fetal calf serum (FCS) and incubated at 37 °C in an atmosphere containing 5% CO_2_. Recombinant retroviruses expressing either pLNCX2-vector or pLNCX2 with the FAM49B gene were generated according to the manufacturer’s instructions (Clontech). MDA-MB-231 or MCF-7 cells were infected with these retroviruses using polybrene (8 μg/mL; Sigma-Aldrich), and expressing cells were then selectively isolated with G418 (750 μg/mL; Calbiochem).

### Immunohistochemistry (IHC)

Immunohistochemical staining was performed as previously described using antibodies against FAM49B, ER, PR, and HER2, and the range and intensity of staining were observed by dewaxing, dehydration, 3%H2O2 elimination of endogenous peroxidase activity, PBS washing, hematoxylin staining, and ethanol and xylene fixation. Immunohistochemical staining range and intensity were assessed using a dual scoring system. According to the previous criteria [8, 9], the expressions of FAM49B, ER and HER2 were evaluated semi-quantitatively. Two independent pathologists conducted separate analyses.

### RNA interference

Recombinant lentiviruses encoding short-hairpin RNAs (shRNAs) specific for human FAM49B, Rab10, and ELAVL1 were designed and prepared by GeneChem (Shanghai, China). The FAM49B target sequence (FAM49B-shRNA#1) used here was 5′-GCAGGCTCTTGCTAAACAGTT-3′; FAM49B-shRNA#2 used was 5′-GCAGCTAATTATGCATTGCAT-3′; FAM49B-shRNA#3 used was 5′-ATCCTGCCATACAGAATGATT-3′;Rab10-shRNA used was 5′-GCCTTCAATACTACCTTTATT-3′; ELAVL1-shRNA used was 5′-GACGATCAAATTCGTTCTCTT-3′; scrambled (scr)- shRNA was used as a negative control, and the target sequence was 5′-TTCTCCGAACGTGTCACGTTT-3′. Lentivirus was added to the cells according to the manufacturer’s recommended protocol. Quantitative real-time polymerase chain reaction (PCR) and western blotting were used to quantify the FAM49B gene knockout rate. Stable cell lines were defined as those with a knockout rate > 80%.

### RNA extraction and quantitative real-time PCR

TRIzol (Invitrogen) was used to extract total RNA and reverse transcribed according to the manufacturer's instructions (Invitrogen). The expression level of FAM49B was detected by real-time fluorescence quantitative PCR. The primers used were as follows. FAM49B: forward, GGCAACTCCAATGCTGAAA; reverse, CACCCACCATTACCCTCAA. GAPDH (used as a control): forward, TGACTTCAACAGCGACACCCA; reverse, CACCCTGTTGCTGTAGCCAAA.

### Co-immunoprecipitation and immunoblotting

The collected cells were lysed with lysis buffer containing 20 mM Tris–HCl pH 7.4, 150 mM NaCl, 1% Triton x-100, and protease inhibitors. Total protein concentration was determined using a bicinchoninic acid protein assay kit (Pierce). Cell extracts (2**–**3 mg) were combined with rabbit anti-FAM49B antibodies for 2 h at 4 °C followed by a second incubation with protein G Plus-agarose for 2 h and subjected to 10% SDS-PAGE followed by transfer to PVDF membranes for immunoblotting. Membranes were blocked in 5% milk/TBS-Tween for 1 h at room temperature. The membranes were incubated overnight with the primary antibody (FAM49B, 1:300, BIOSS; Rab10, 1:1000, Abcam; TLR4, 1:1000, Abcam; ELAVL1, 1:500, Abcam) at 4 °C, followed by incubation with the secondary antibody for 2 h at room temperature. Proteins were visualized using enhanced chemiluminescence (Amersham), and the results were quantified using ImageJ (NIH).

### MTT assay

The culture medium containing 10% fetal calf serum was mixed into single cell suspension, and about 1.5 × 10^4^ cells /mL were inoculated on the 96-well plate. After 2–5 days of culture, 10 mol/L MTT (5 mg/mL) was added to each well. After 4 h incubation, the culture was terminated, and the culture supernatant in the well was carefully absorbed and discarded. For suspension cells, centrifugation was required to absorb and discard the culture supernatant in the well, then 100 mol/L DMSO was added to each well, and the absorbance was measured at 490 nm with a microplate reader. Each set uses three duplicate holes.

### Wound healing assay

The experiment was performed as recommended by the manufacturer. The cells were grown in DMEM medium containing 10%FBS, followed by a 24-well cell culture plate with 2.0 × 10^5^ cells per well. After 24 h of cell growth, the single-layer fusion was observed at 70–80%. A new 1 ml spear head was used to gently and slowly scratch the cells in the monolayer culture, and the scratches traversed through the holes and formed a straight line in the same direction. Then vertical to the first scratch, make the second scratch, each hole of the scratch are cross cross type; After the scratch, in order to remove the shedding cells, the plate hole was slowly and gently cleaned with the medium twice; After that, fresh medium was added to each well and the cells were allowed to grow for 48 h. The cells were washed twice with PBS, and then fixed with 3.7% paraformaldehyde for 30 min and stained with 0.1% crystal violet for 30 min. Wound images were obtained at 0, 8 and 24 h after implant removal. Movement rate is calculated as: movement distance/initial width of wound field.

### Transwell assay

The transwell chamber is composed of 24-well tissue culture plate and 12 well cell culture insert, which contains 8 µm pore size of polycarbonate membrane. Serum-free cell suspensions were prepared and counted at a density of 5 × 10^4^ cells/well (24-well plate). The upper culture medium was carefully removed, and 100 µL cell suspension was added. In the lower cavity, 600 M of 30% FBS medium was added. The mixture was incubated at 37 °C for 24 h. The cells were fixed with 4% paraformaldehyde for 30 min. Then, 1**–**2 drops of stain were added and the cells were transferred to the submembrane surface for 1**–**3 min. Photographs were taken under a microscope. In each transwell chamber, the 6 fields were randomly selected. The cells were counted with 200× photos, and the data were analyzed to compare the difference of cell transfer ability between the experimental group and the control group.

### Tumor growth and metastasis in nude mice

Female BALB/c nude mice (4**–**6 weeks old) were purchased from Shanghai Lingchang Biological Technology Ltd. (Shanghai, China). MDA-MB-231 cells (1 × 10^7^) transfected with FAM49B-shRNA or scr-shRNA were implanted subcutaneously into the flanks of BALB/c female nude mice according to previously described criteria [12, 14]. The nude mice were sacrificed by injecting excessive 2% Pentobarbital Sodium 9 weeks later, and the tumors were weighed.

For the in vivo metastasis experiment, shFAM49B- or scr-shRNA-expressing MDA-MB-231 cells were injected into the caudal vein of nude mice under anesthesia by inhalation of a 1:1 mixture of isoflurane gas and oxygen. The nude mice were sacrificed by injecting excessive 2% Pentobarbital Sodium 10 weeks later, and their metastatic lung nodules were quantified.

For the in vivo chemoresistance experiment, shFAM49B- MDA-MB-231 cells were injected into the flanks of nude mice (10 mice/group). After two weeks, each group was divided randomly into two subgroups that were either left untreated or received intraperitoneal injections of doxorubicin (4 mg kg^−1^) every 5 days (three cycles), as previously described by Ghebeh et al. [15]. The nude mice were sacrificed by injecting excessive 2% Pentobarbital Sodium 8 weeks later, and the tumors were weighed. Animal handling and research protocols were approved by the Ethics Committee of the Affiliated Hospital of Hebei Engineering University.

### Cell apoptosis analysis

After infection, the cell culture supernatant of each experimental group was collected in a 5 mL centrifuge tube, the cells were washed by D-Hanks once, the cells were digested by trypsin. 1500rmp for 5 min centrifugation and discard the supernatant. The cells were washed with PBS, precipitated once, centrifuged at 1500rmp for 5 min. The cells were washed with binding buffer, precipitated once, centrifuged at 1500 rmp for 5 min. Take 100 uL of cell suspension (1 × 10^5^–1 × 10^6^ cells), the cells were stained with annexin V and propidium iodide (PI) using the annexin V-PI detection kit (Roche, Mannheim, Germany). Apoptosis was measured using flow cytometry (BD Biosciences, San Jose, CA, USA).

### Microarray detection and data analysis

Total RNA was extracted from MDA-MB-231 cells treated with the TRIzol reagent. MDA-MB-231 cells were treated with lentiviral vectors of SCR-shrNA or Fam49B-shrNA. RNA was evaluated using NanoDrop 2000 and Agilent 2100. Subsequent experiments were conducted using these samples. Both cDNA strands were synthesized via reverse transcription. Next, labeled cRNA was synthesized by in vitro transcription using the GeneChip 3IVT Expression Kit. GeneChip Hybridization Wash and Stain Kit was used for hybridization, washing, and staining. GeneChip Scanner 3000 was used to scan the arrays for evaluation of data. Gene expression profiling was performed using the Affymetrix Human Gene 1.0 ST platform. The criteria used to determine the genes differentially expressed between MDA-MB-231 cells infected with FAM49B-shRNAs and scr-shRNAs were p < 0.05 and |FC|> 1.5. The Innovative Pathway Analysis (IPA) database was used for pathway analysis, disease analysis, functional analysis, network analysis, and downstream gene analysis.

### ONCOMINE analysis

The mRNA levels of FAM49B in BC samples (and normal control samples) were determined using the ONCOMINE database (www.oncomine.org), a publicly accessible online cancer microarray database designed to facilitate discovery from genome-wide expression analyses. In this study, Student’s *t*-test was used to generate a p-value for comparing cancer specimens and normal control datasets. The fold change was defined as 2, and the p-value was set at 0.01.

### Kaplan–Meier plotter survival analysis

Prognostic values of FAM49B expression in BC samples were assessed by displaying the disease-free survival (DFS) and overall survival (OS) using the Kaplan–Meier plotter (http://kmplot.com/analysis/) [16]. Kaplan–Meier survival curve, log-rank p-value, and hazard ratio (HR) with a 95% confidence interval (CI) were calculated and plotted in R using Bioconductor packages.

### Statistical analysis

Student’s *t*-test was used to analyze the real-time PCR, western blotting, MTT, and migration assays. χ^2^ analysis was used to assess whether and to what extent FAM49B expression was correlated with tumor size, histological grade, and lymph node metastasis rate. Statistical significance of differences was considered to be at a p-value less than 0.05.

## Results

### FAM49B mRNA levels are elevated in BC and correlate with poor prognosis

**Fig. 1:**
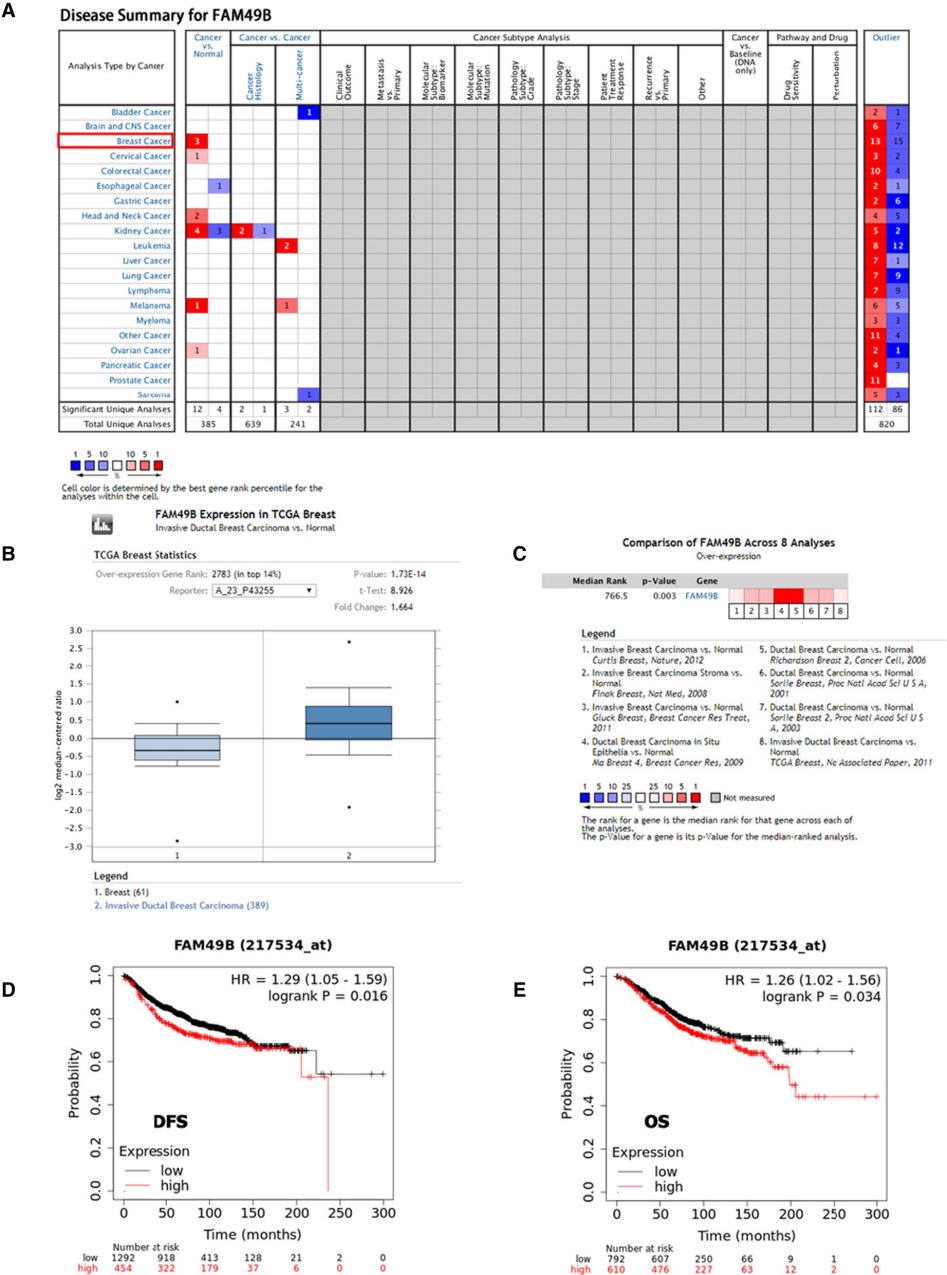
Analysis of FAM49B mRNA expression and prognostic values in BC. **A** FAM49B mRNA expression (shown in red frame) in normal and BC tissues, as per ONCOMINE. **B** FAM49B mRNA expression, as per TCGA database. **C** Meta-analysis of gene expression profiling for FAM49B in BC using ONCOMINE, with *p* < 0.05 and fold change > 1.5. The colored squares indicate the median rank for FAM49B across each analysis comparing BC tissue with normal tissue. **D**, **E** High FAM49B mRNA levels were correlated with reductions in DFS (**D**) and OS (**E**)

FAM49B expression has also been observed in several human cancers (Fig. 1A). ONCOMINE analysis revealed that FAM49B mRNA expression was significantly higher in BC samples than in normal samples across a wide variety of datasets covering different types of BC. FAM49B transcripts were elevated 1.664-fold in BC samples compared to normal tissues, in a dataset containing 450 samples derived from The Cancer Genome Atlas (TCGA) database (Fig. 1B). The pooled results of the eight clinical cohorts showed a significant increase in FAM49B expression in BC (*p* = 0.003, Fig. 1C). Thereafter, we assessed the prognostic effect of FAM49B in BC using the Kaplan–Meier survival analysis plot. This revealed that high FAM49B mRNA expression was correlated with reduced DFS and OS in all BC patients (HR = 1.29, *p* = 0.016; HR = 1.26, *p* = 0.034, respectively, Fig. 1D, E).

### FAM49B protein expression is elevated in BC tissues and correlates with poor prognosis

**Fig. 2:**
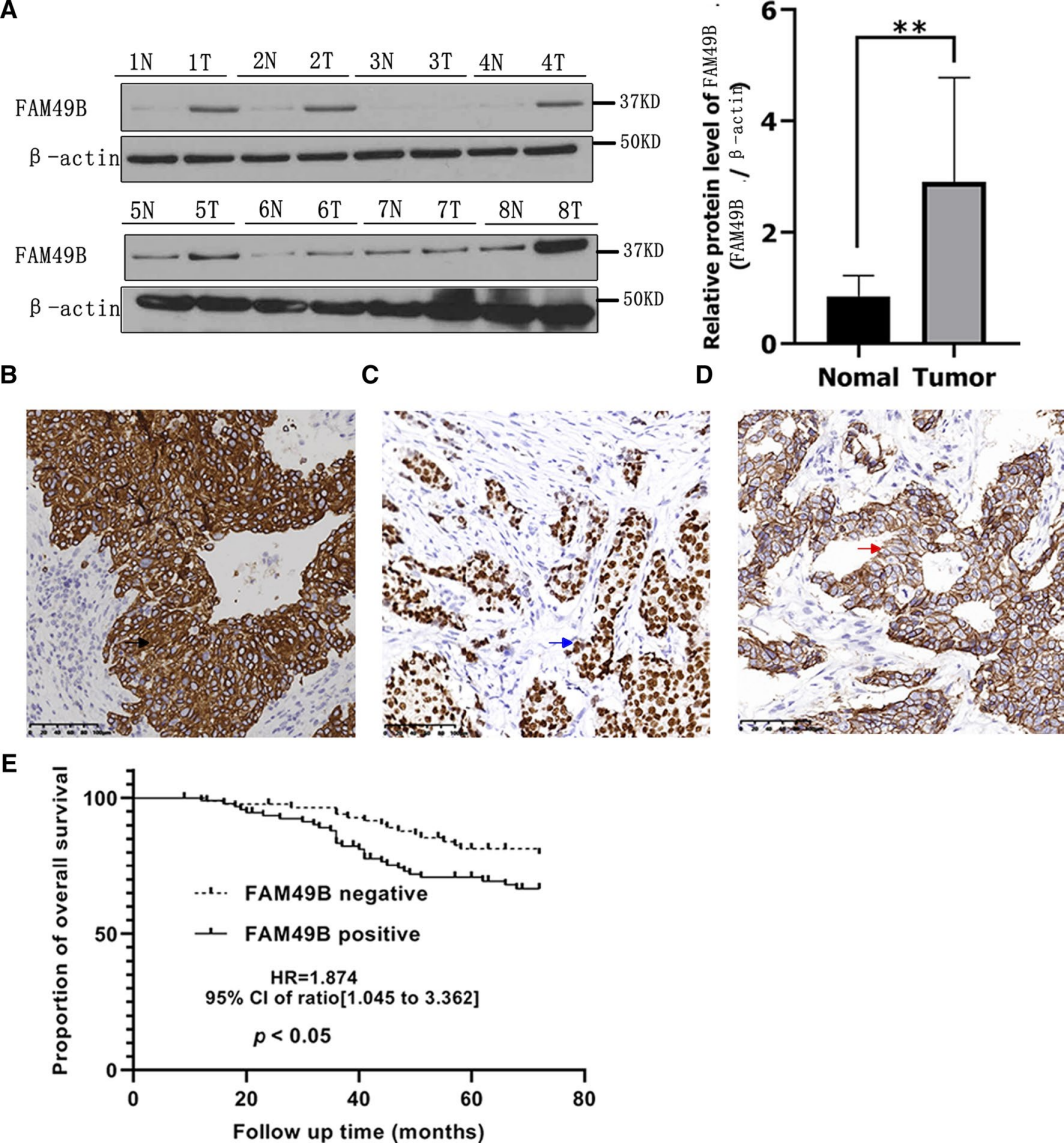
High expression of FAM49B in human BC. **A** Expression status of FAM49B in matched normal (N) and cancerous (T) regions isolated from BC patients following western blotting. Expression levels of FAM49B, ER, and HER2, as per immunohistochemical staining (**B**–**D**). Blue arrow represents staining in the nucleus; black arrow represents staining in the cytoplasm; red arrow represents staining in the cytomembrane. **E** Kaplan–Meier survival curves showing survival in 180 BC patients, with respect to FAM49B expression. Results are presented as the mean ± SD. The statistical significance was assessed by Student’s *t*-test; **p < 0.01

The expression of FAM49B protein in eight BC tissues and eight adjacent non-tumor tissues was assessed using western blotting. As shown in Fig. 2A, FAM49B protein expression levels were significantly higher in BC tissues than in normal breast tissues (*p* < 0.01).

Next, we performed IHC to visualize FAM49B protein expression in BC tissues. This revealed that FAM49B staining occurred mainly in the cytoplasm of BC samples. ER staining was observed in the nucleus of BC cells, whereas HER2 staining was localized in the cellular membrane. Examples of positive protein expression of FAM49B, ER, and HER2 are shown in Fig. 2B–D. IHC analysis showed that the positive rate of FAM49B expression in cancer tissue samples was 52.8% (95/180 cases). The positive rate was significantly higher than that of the adjacent normal breast tissue (28.9%; 26/90 samples) (*p* = 0.000, Table 1). In addition, positive FAM49B protein expression was positively correlated with tumor size, histological grade, and lymph node metastasis of BC (*p* = 0.006, *p* = 0.013, and *p* = 0.023, respectively, Table 1).

**Table 1:** The relationship between FAM49B expression and the clinicopathological factors (n = 180)

Variable	n	FAM49B^−^	FAM49B^+^	*p* varible
Tissue				0.000
Cancer tissue	180	85	95	
Adjacent tissue	90	64	26	
Age				0.927
≥ 40	152	72	80	
< 40	28	13	15	
Tumor size				0.006
T1	38	25	13	
T2	112	52	60	
T3	30	8	22	
Histological grades				0.013
I	20	12	8	
II	65	38	27	
III	95	35	60	
Lymph node metastasis				0.023
Negative	67	39	28	
Positive	113	46	67	

“+”, positive; “−”, negative

Correlation analysis showed that the FAM49B-positive expression rate was significantly higher in ER-negative (ER-) cases than in ER-positive (ER +) cases (*p* = 0.007, Table 2). Conversely, FAM49B-positivity was significantly higher in HER2+ cases than in HER2- cases (*p* = 0.011, Table 2). However, there was no significant difference in FAM49B expression with regard to PR positivity (*p* = 0.071, Table 2).

**Table 2:** Correlations between FAM49B expression and immunohistochemical markers

Variable	n	FAM49B^−^	FAM49B^+^	*p* varible
ER				0.007
−	58	19	39	
+	122	66	56	
PR				0.071
−	64	29	45	
+	116	56	50	
HER2				0.011
−	137	72	65	
+	43	13	30	

“+”, positive; “−”, negative

*ER* estrogen receptor, *HER2* human epidermal growth factor receptor 2, *PR* progesterone receptor

Furthermore, our patient follow-up analysis showed that 45 of 180 patients died, and the 6-year overall survival rate was 85.0%. FAM49B expression was positive in the BC samples of 29 out of the 45 patients that died, while only 16 cases of death occurred in the group with negative FAM49B expression. Kaplan–Meier analysis showed that compared with FAM49B-negative BC patients, the survival rate of FAM49B-positive BC patients was significantly reduced (log-rank test, *p* < 0.05, HR = 1.874, 95% CI 1.045–3.362, Fig. 2E).

### FAM49B promotes the proliferation and migration of BC cells in vitro

**Fig. 3:**
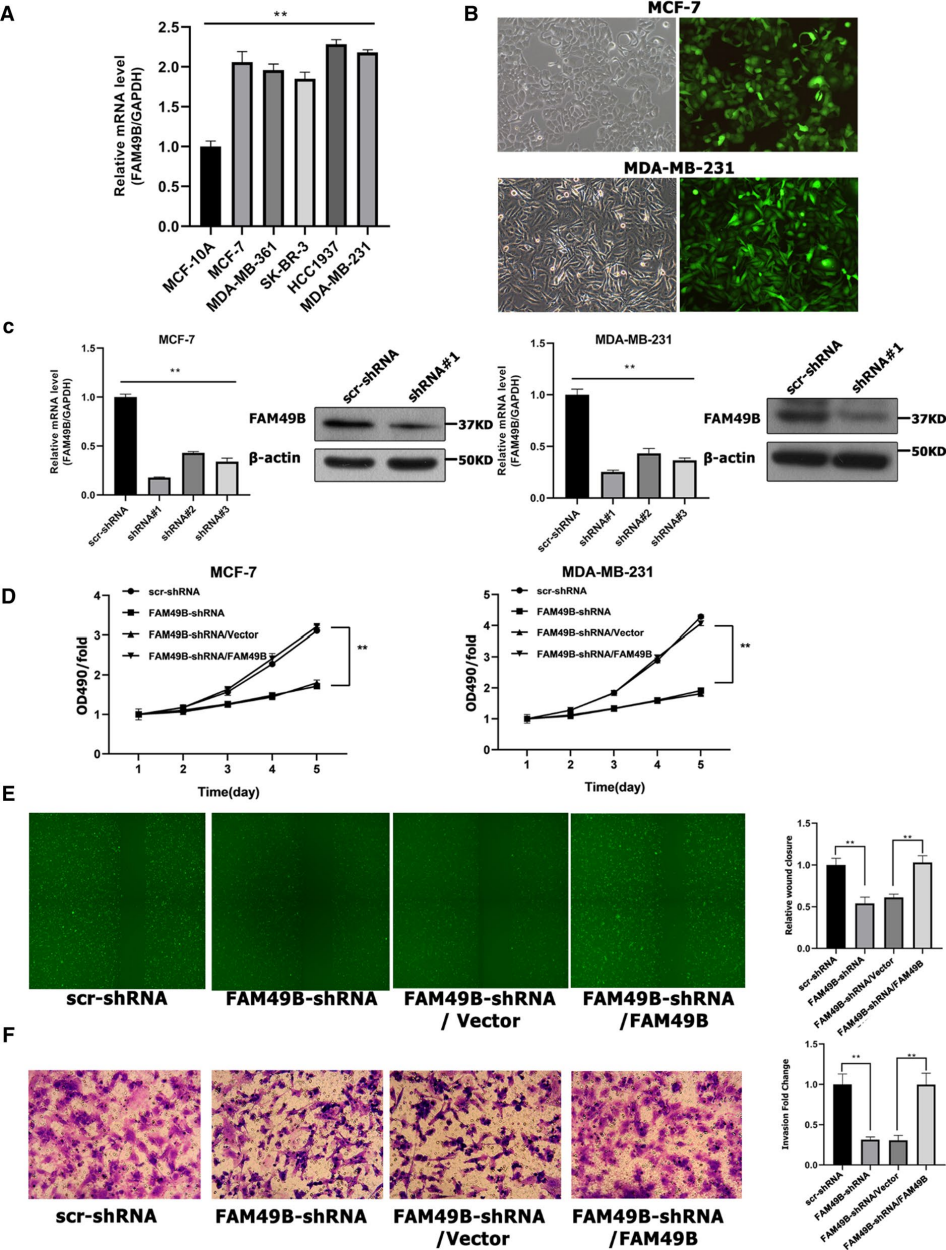
Effects of FAM49B on proliferation and migration in vitro. **A** Levels of FAM49B mRNA were analyzed by real time-PCR in five BC cell lines (n = 3). **B** MCF-7 and MDA-MB-231 cells infected with FAM49B-shRNA lentivirus were examined by fluorescence microscopy, 3 days after infection. **C** FAM49B mRNA and protein expression levels were examined by real time-PCR and western blotting in MCF-7 and MDA-MB-231 cells infected with FAM49B shRNA (n = 3). pLNCX2-FAM49B or pLNCX2-vector were transfected into FAM49B-shRNA-expressing MCF-7 or MDA-MB-231 cells. Cell growth rate (**D**) was monitored by MTT assay in MCF-7 and MDA-MB-231 cells; **E**, **F** Cell migration was examined by wound healing assay (**E**) and transwell assay (**F**) in MDA-MB-231 cells (n = 3). Results are presented as the mean ± SD. The statistical significance was assessed by Student’s *t*-test; **p < 0.01

First, FAM49B mRNA expression levels in BC cell lines were evaluated using real-time PCR, which showed that FAM49B expression levels were significantly higher in the five BC cell lines—MDA-MB-231, MDA-MB-361, HCC1937, MCF-7, and SKBR-3—than in the non-tumorigenic breast epithelial cell line MCF-10A (*p* < 0.01; Fig. 3A). However, FAM49B expression was highly expressed in these 5 BC cells. MDA-MB-231 and MCF-7 cell lines were selected for subsequent knockdown or overexpression studies.

We used shRNA to knock out FAM49B in MCF-7 and MDA-MB-231 BC cells and confirmed that the infection efficiency of FAM49B-shRNA and scr-shRNA exceeded 80% at 3 days after infection (Fig. 3B). The results of western blotting and real-time PCR showed that the levels of FAM49B protein and mRNA in FAM49B knockout cells were lower than those in scr-shRNA control cells (*p* < 0.01, Fig. 3C). FAM49B-shRNA#1 was selected for subsequent analyses. The MTT analysis method was used to analyze the proliferation rate of MCF-7 and MDA-MB-231 cells. MCF-7 and MDA-MB-231 cells were infected with FAM49B-shRNA or scr-shRNA. Within 5 days of FAM49B downregulation, the number of cells decreased and the cell proliferation rate was significantly reduced, as assessed by MTT analysis (*p* < 0.01, Fig. 3D). However, re-expression of FAM49B in the two FAM49B-shRNA BC cell lines completely restored cell proliferation (*p* < 0.01, Fig. 3D).

Furthermore, wound healing and transwell assays were performed to evaluate the effect of FAM49B on BC cell migration. MDA-MB-231 cells with downregulated FAM49B migrated much slower than scr-shRNA control cells, indicating that the inhibitory effect of FAM49B effectively inhibited cell migration (*p* < 0.01, Fig. 3E, F). However, returning the FAM49B expression ability back to the FAM49B-shRNA BC cell line completely restored cell migration (*p* < 0.01, Fig. 3E, F). Knockdown of FAM49B inhibits tumor cell growth and metastasis in vitro. Therefore, FAM49B plays a significant role in cancer growth and metastasis in vitro.

**Fig. 4:**
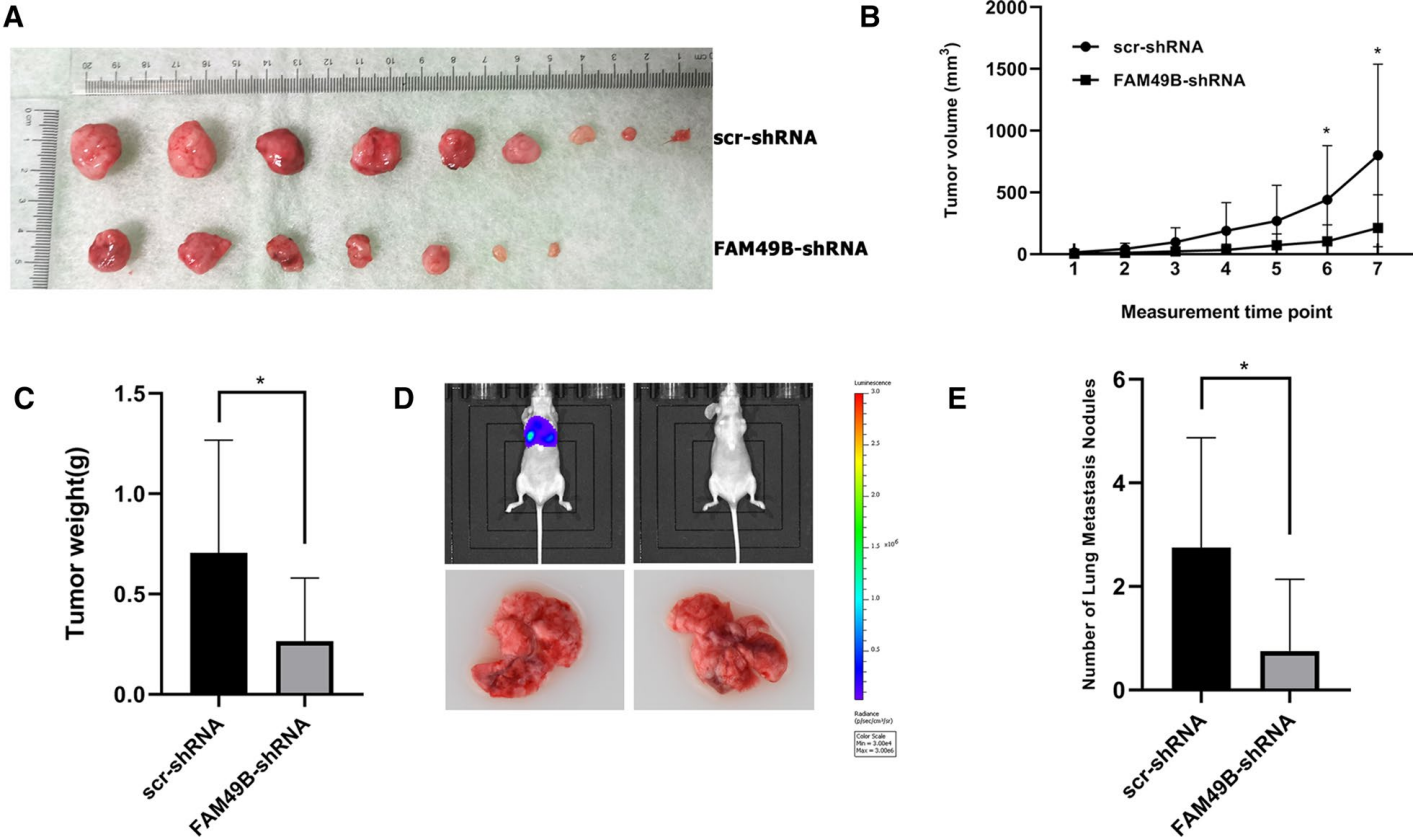
Effects of FAM49B on proliferation and migration in vivo. **A**–**C** scr-shRNA- and FAM49B-shRNA- MDA-MB-231 cells were injected into the flanks of nude mice. **A** Tumor growth and **B** tumor volume were measured on the indicated days. **C** Tumor weights were measured after mice were sacrificed. **D**, **E** shFAM49B-MDA-MB-231 cells and control cells were injected into the caudal vein of nude mice. The mice were sacrificed, and their metastatic lung nodules were quantified. Results are presented as the mean ± SD. The statistical significance was assessed by Student’s *t*-test; *p < 0.05

We investigated whether FAM49B could regulate the growth capacity of BC cells in vivo. MDA-MB-231 cells expressing scr-shRNA or FAM49B-shRNA were implanted into nude mice (n = 10), and tumor progression was monitored for 7 weeks, after which the mice were sacrificed. Compared with the scr-shRNA control group, the volume of MDA-MB-231 tumors expressing FAM49B-shRNA was significantly reduced (*p* < 0.05, Fig. 4A, B). At the end of the observation period, the tumors of FAM49B-shRNA MDA-MB-231 were significantly reduced in weight compared with the control group (*p* < 0.05, Fig. 4C).

To test whether FAM49B regulates metastatic potential in vivo, this study quantified lung metastatic nodules following injection of MDA-MB-231-shFAM49B and their corresponding control cells into the caudal vein of nude mice. Compared with control mice, the lung metastasis nodules of mice injected with MDA-MB-231-shFAM49B cells were significantly reduced (*p* < 0.05, Fig. 4D, E). These results indicate that FAM49B also plays a role in cancer growth and metastasis in vivo.

### FAM49B regulates expression of BC genes

**Fig. 5:**
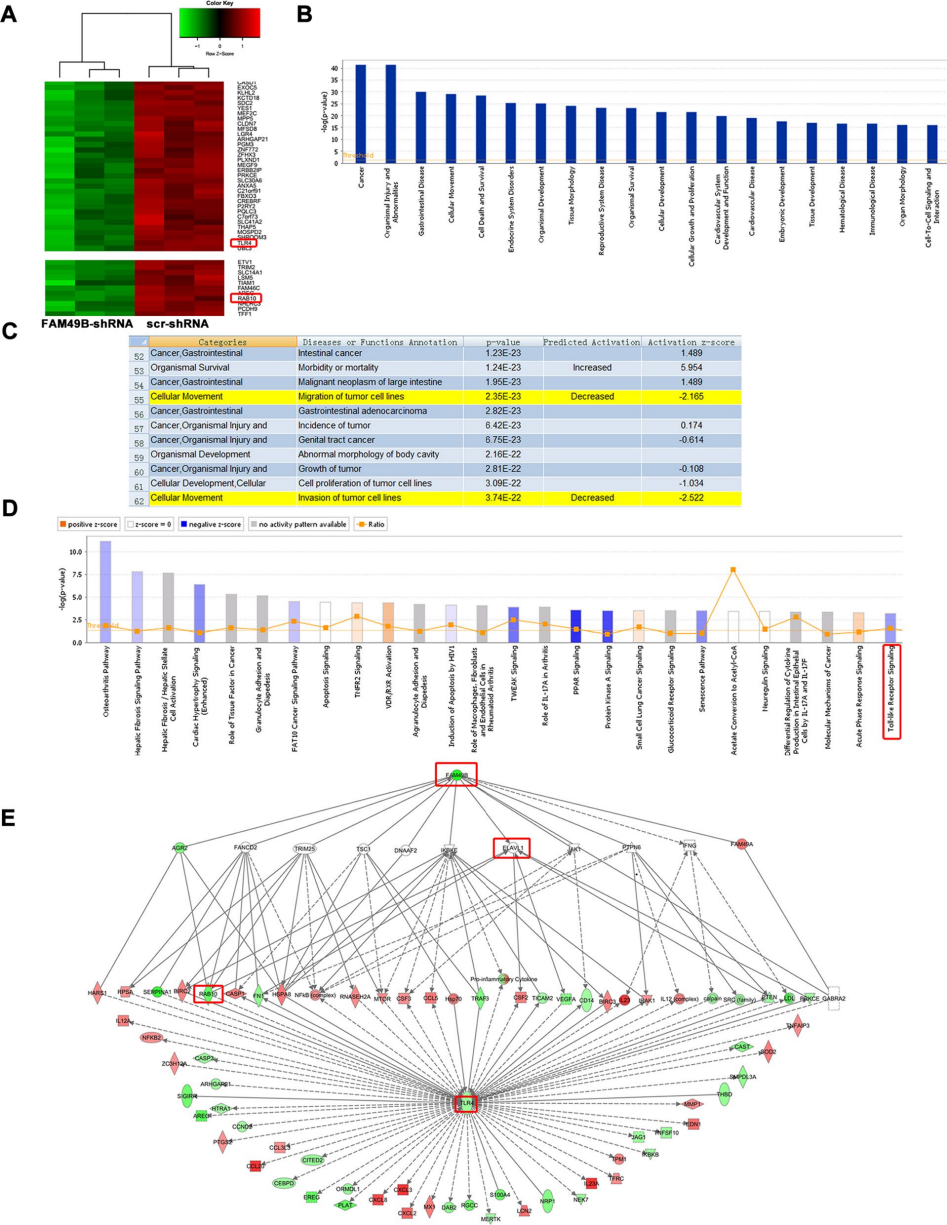
Changes in gene expressions in MDA-MB-231 cells with FAM49B knockdown, analyzed using microarray. **A** Heat-map analysis showing 1063 genes that were detected as being altered, by microarray profiling. *p* < 0.05 and |fold Change|≥ 1.5. **B**, **C** Disease and function enrichment of whole-genome expression microarray in FAM49B knockdown MDA-MB-231 cells was performed using IPA. **D** Canonical pathway enrichment of whole-genome expression microarray in FAM49B knockdown MDA-MB-231 cells was performed using IPA. **E** Gene interaction network of FAM49B and related genes in Toll-like receptor signaling pathway. The results show that Rab10, TLR4, and ELAVL1 (shown in red frame) were located in the central regulatory position. Data are shown as mean ± SD

To clarify the mechanism by which FAM49B plays a role in BC, we performed a genome-wide expression microarray on MDA-MB-231 cells expressing scr-shRNA or FAM49B-shRNA. Consequently, we detected 1063 genes that showed differential expression (|fold change|≥ 1.5 and *p* < 0.05), including 393 upregulated genes and 670 downregulated genes (Fig. 5A). Using on the IPA database, FAM49B knockdown was found to affect the expression of related genes, such as cancer, cell movement, and cell death and survival (Fig. 5B). Knockdown of FAM49B significantly inhibited tumor cell migration and invasion of tumor cells (Fig. 5C). In addition, FAM49B knockdown had a significant inhibitory effect on several key cancer pathways, such as TWEAK, PPAR, and Toll-like receptor signaling pathways (Fig. 5D), indicating that FAM49B can regulate the malignant phenotype of BC.

### FAM49B promoted BC cell proliferation and migration by upregulating Rab10/TLR4 pathway

**Fig. 6:**
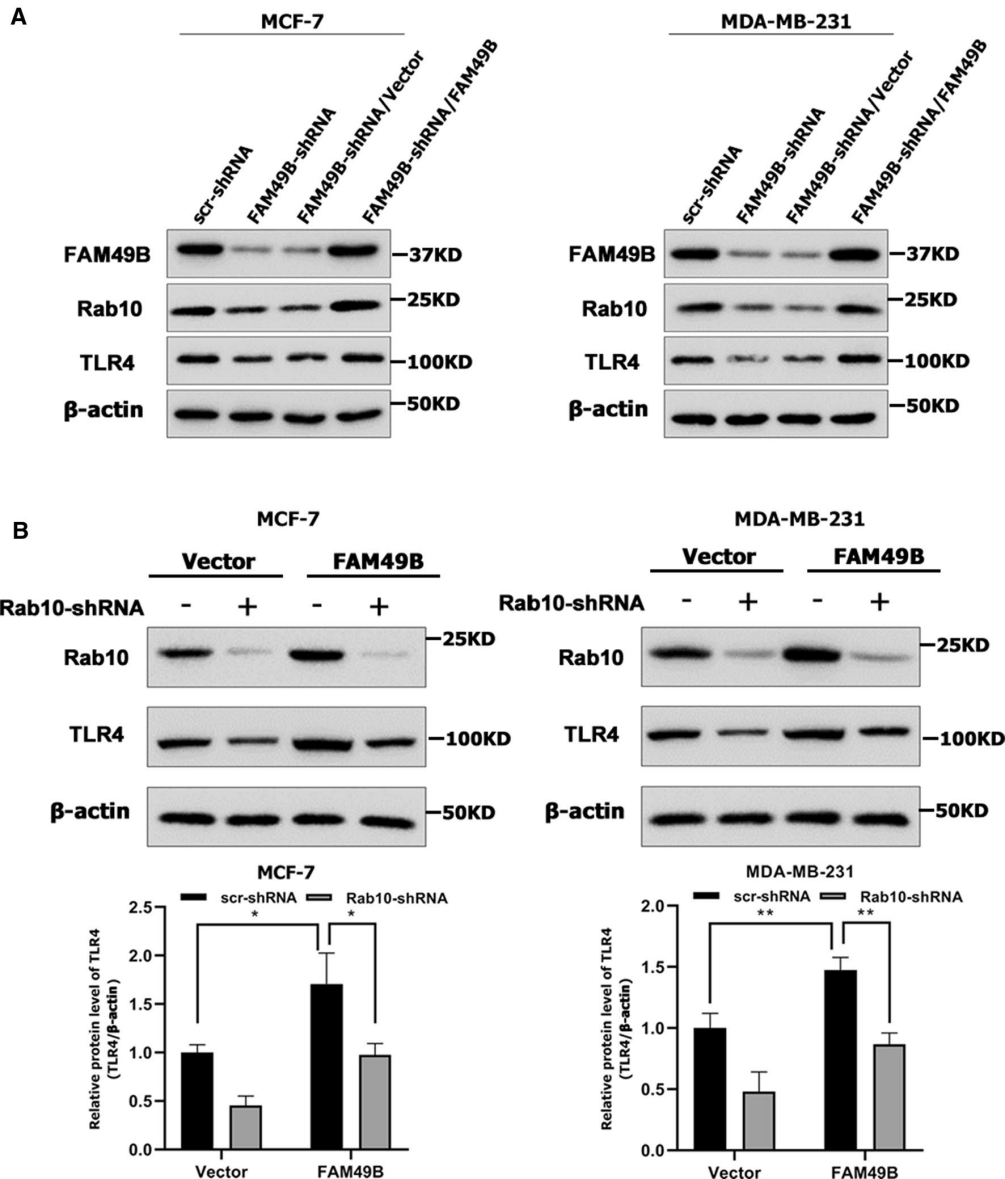
FAM49B positively regulates Rab10 and TLR4 expression. **A** pLNCX2-FAM49B or pLNCX2-vector were transfected into FAM49B-shRNA-expressing MCF-7 or MDA-MB-231 cells. FAM49B, Rab10, and TLR4 expression was quantified via western blotting (n = 3). **B** scr- or Rab10-shRNA were transfected into FAM49B-overexpressing MCF-7 and MDA-MB-231 cells. Rab10 and TLR4 expression was quantified via western blotting (n = 3). Results are presented as the mean ± SD. The statistical significance was assessed by Student’s *t*-test; *p < 0.05, **p < 0.01

According to the IPA database, the expression of Rab10 and Toll-like receptor 4 (TLR4) mRNA was inhibited when FAM49B was silenced in Toll-like receptor signaling and TLR4 may be the downstream target of Rab10 (Fig. 5A, E). Rab10 can accelerate the transport of TLR4 to the plasma membrane. Rab10 knockout reduced the expression of membrane TLR4 and reduced the production of inflammatory factors induced by LPS [17]. To further study the regulatory mechanism between FAM49B, Rab10, and TLR4 in BC, FAM49B knockdown in the BC cell lines MCF-7 and MDA-MB-231 was performed using shRNA, and we found that FAM49B knockdown significantly inhibited the protein expression of Rab10 and TLR4 in BC cell lines. However, re-expression of FAM49B back into the two FAM49B-shRNA BC cell lines completely restored Rab10 and TLR4 expression (*p* < 0.01, Fig. 6A), indicating that FAM49B positively regulates Rab10 and TLR4 expression in BC cells.

To identify whether Rab10 is a key factor in this pathway, endogenous Rab10 was silenced in FAM49B-transfected MCF-7 and MDA-MB-231 cells. BC cells transfected with a non-functional vector were used as controls. It was found that Rab10 could inhibit protein expression of TLR4 in the control group and FAM49B upregulated protein expression of TLR4 in the FAM49B overexpression group (*p* < 0.05, Fig. 6B). However, TLR4 expression was significantly decreased by silencing Rab10 in the FAM49B overexpression group (*p* < 0.05, Fig. 6B). These results suggest that Rab10 positively regulates TLR4 expression in BC cells and is required in the FAM49B/TLR4 pathway.

**Fig. 7:**
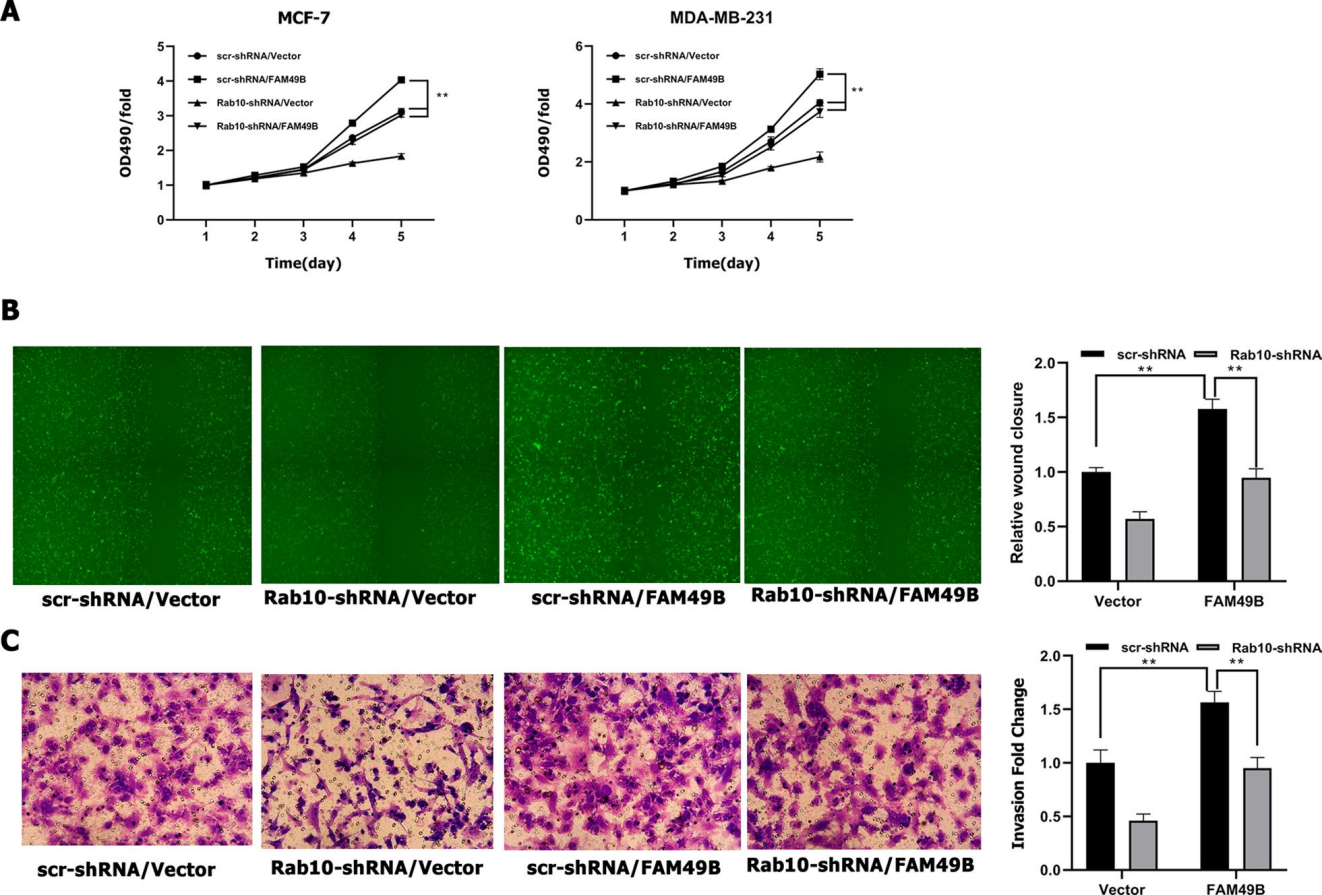
FAM49B regulates proliferation and migration of BC cells by upregulating Rab10. scr- or Rab10-shRNA were transfected into FAM49B-overexpressing MCF-7 and MDA-MB-231 cells. Cell growth rate (**A**) was monitored by MTT assay in MCF-7 and MDA-MB-231 cells (n = 3). **B**, **C** Cell migration was examined via wound healing (**B**) and transwell assays (**C**) in MDA-MB-231 cells (n = 3). Results are presented as the mean ± SD. The statistical significance was assessed by Student’s *t*-test; **p < 0.01

To verify whether FAM49B promotes the proliferation and migration of BC cells through Rab10 regulation, Rab10 was knocked down in FAM49B-transfected MDA-MB-231 and MCF-7 cells. We already knew that proliferation and migration were promoted in FAM49B overexpressing cells, as assessed by MTT assay, wound healing assay, and transwell assay (*p* < 0.01, Fig. 7A–C). However, the promotion of proliferation and migration mediated by FAM49B overexpression was reversed by Rab10 knockdown (*p* < 0.01, Fig. 7A–C). These observations demonstrate that Rab10 is required for the FAM49B pathway-mediated migration and proliferation of BC cells.

### FAM49B positively regulates Rab10/TLR4 pathway by stabilizing ELAVL1 protein

**Fig. 8:**
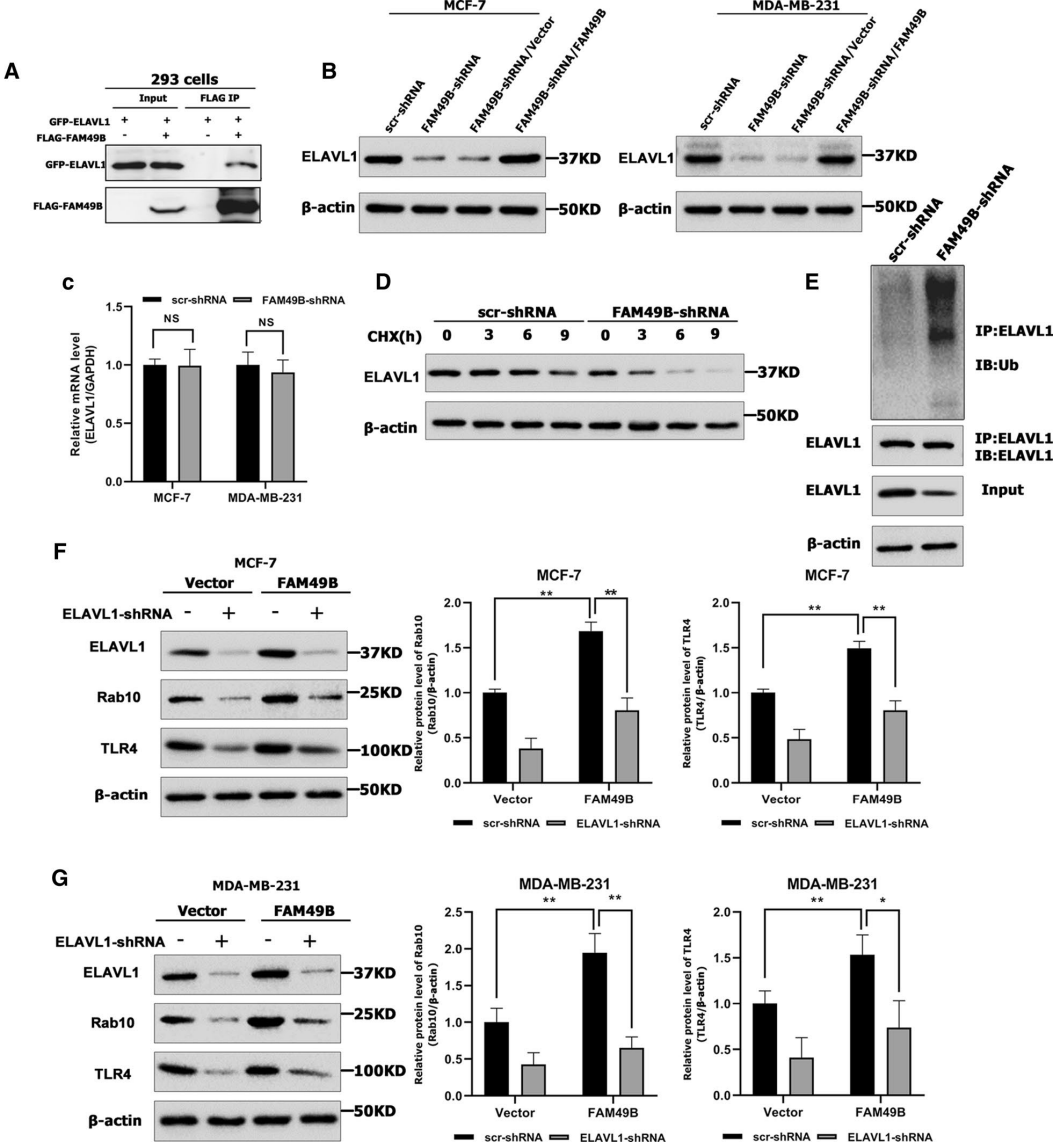
FAM49B positively regulates Rab10/TLR4 pathway by stabilizing ELAVL1. **A** Exogenous interaction between FAM49B and ELAVL1 in 293 cells, observed via co-immunoprecipitation. **B** pLNCX2-FAM49B or pLNCX2-vector were transfected into FAM49B-shRNA-expressing MCF-7 or MDA-MB-231 cells. ELAVL1 expression was quantified via western blotting (n = 3). **C** Level of ELAVL1 mRNA was determined using quantitative real-time PCR in FAM49B-shRNA-expressing MCF-7 or MDA-MB-231 cells. **D** FAM49B mediated ELAVL1 stabilization in BC cells. scr- or FAM49B-shRNA-expressing MDA-MB-231 cells were treated with CHX (50 μM) for the indicated time points and analyzed for endogenous ELAVL1 expression via western blotting. **E** FAM49B inhibition increased ELAVL1 ubiquitination in MDA-MB-231 cells. HA-Ubiquitin was electroporated into scr- or FAM49B-shRNA-expressing MDA-MB-231 cells. Cells were treated with MG132 (20 μM) for 2 h. ELAVL1 complex in resulting lysates was examined using an antibody against Ubiquitin (Ub). **F**, **G** scr- or ELAVL1-shRNA were transfected into FAM49B-overexpressing MCF-7 and MDA-MB-231 cells. ELAVL1, Rab10, and TLR4 expression was quantified via western blotting (n = 3). Results are presented as the mean ± SD. The statistical significance was assessed by Student’s *t*-test; *p < 0.05, **p < 0.01

According to the IPA database, ELAVL1 may be the downstream target of FAM49B and plays a central role in regulating the Rab10/TLR4 pathway (Fig. 5E). ELAV-like RNA binding protein 1 (ELAVL1) is a member of the ELAVL family of RNA-binding proteins that contain several RNA recognition motifs, and it selectively binds AU-rich elements (AREs) found in the 3′ untranslated regions of mRNAs. It is highly expressed in many cancers and could be potentially useful in cancer diagnosis, prognosis, and therapy [18–23]. To identify target proteins downstream of FAM49B, the Pathway Commons Protein–Protein Interactions dataset (http://amp.pharm.mssm.edu/Harmonizome/) was used. This dataset identified ELAVL1 as a potential interactor of FAM49B [24]. Thereafter, we performed a co-immunoprecipitation assay and found that exogenous FAM49B interacted with ELAVL1 in 293 cells (Fig. 8A). Moreover, FAM49B knockdown reduced protein expression of ELAVL1 in MCF-7 and MDA-MB-231 BC cells (*p* < 0.01, Fig. 8B). Co-transfection of FAM49B into FAM49B-shRNA BC cells completely restored ELAVL1 expression (*p* < 0.01, Fig. 8B). In addition, FAM49B knockdown did not alter ELAVL1 mRNA expression (Fig. 8C). Therefore, FAM49B may regulate ELAVL1 protein expression at the posttranslational level. To test this hypothesis, we determined whether FAM49B maintained ELAVL1 stability by treating MDA-MB-231 cells with cycloheximide (CHX) to inhibit protein synthesis. The downregulation of FAM49B induced ELAVL1 degradation in MDA-MB-231 cells (Fig. 8D), suggesting that FAM49B stabilizes ELAVL1 in BC cells. Moreover, FAM49B knockdown promoted ELAVL1 ubiquitination (Fig. 8E).

To further understand the role of ELAVL1 in this pathway, endogenous ELAVL1 was silenced in FAM49B-transfected MCF-7 and MDA-MB-231 cells. BC cells transfected with a non-functional vector were used as controls. We found that ELAVL1 could inhibit protein expression of Rab10 and TLR4 in the control group and FAM49B upregulated protein expression of Rab10 and TLR4 in the FAM49B overexpression group (*p* < 0.01, Fig. 8F, G). However, Rab10 and TLR4 expression was significantly decreased by silencing ELAVL1 in the FAM49B overexpression group (*p* < 0.01, Fig. 8F, G). These results suggest that ELAVL1 positively regulates Rab10 and TLR4 expression in BC cells and is required in the FAM49B pathway.

### FAM49B promotes anthracycline resistance to chemotherapy in triple-negative BC (TNBC) cells by targeting the ELAVL1/Rab10/TLR4/NF-κB signaling pathway

**Fig. 9:**
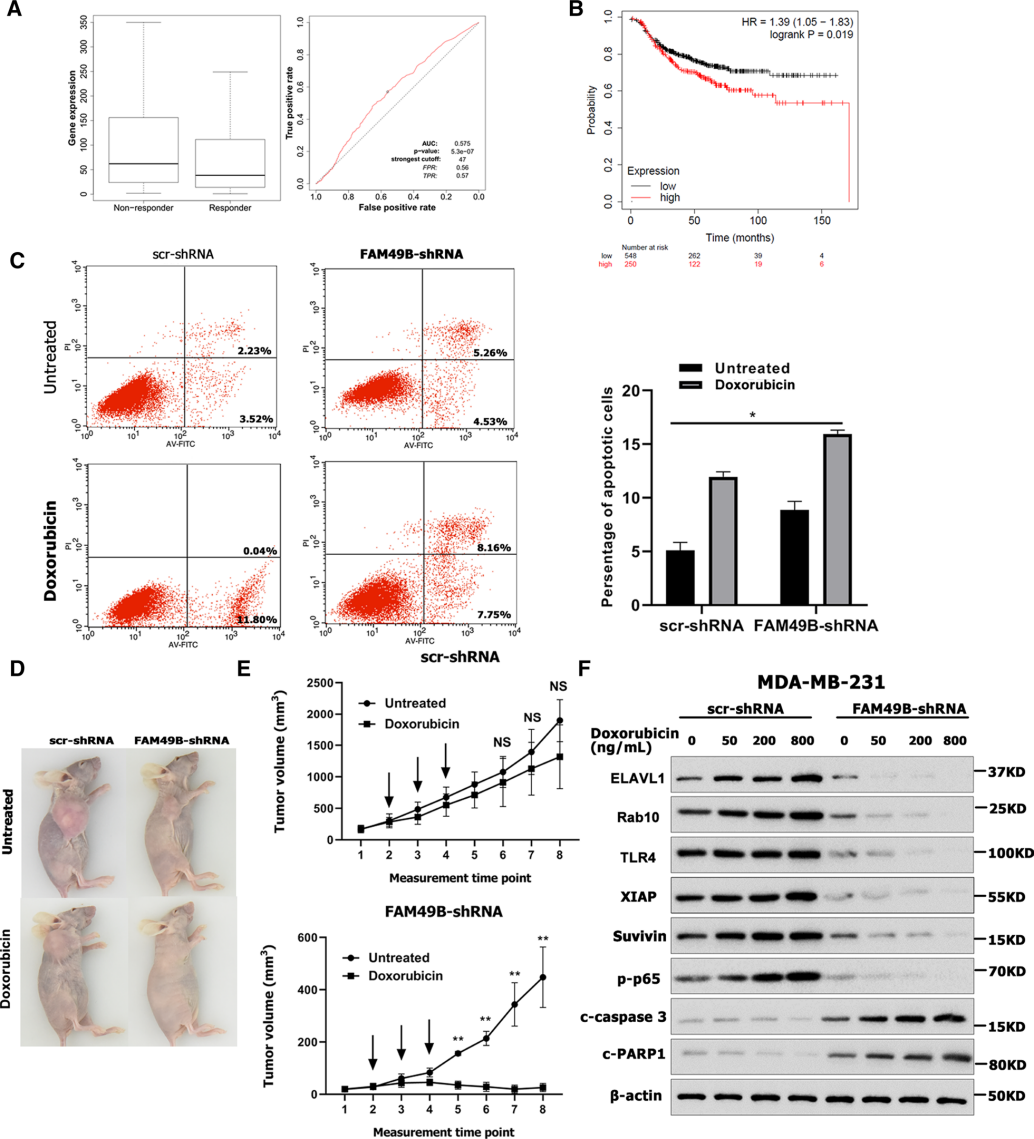
FAM49B-knockdown BC cells are more sensitive to doxorubicin. **A** FAM49B mRNA expression was lower in anthracycline-sensitive BC samples, as per ROC Plotter analysis. **B** High FAM49B mRNA level was associated with decreased RFS in BC patients who had received chemotherapy. **C** The dot matrix of Annexin V/PI double staining was generated after scr- and FAM49B-shRNA MDA-MB-231 cells were treated or not treated with 200 ng/mL of doxorubicin for 24 h (n = 3). scr- and FAM49B-shRNA MDA-MB-231 cells were injected into mice, as described previously. Tumor growth (**D**) and tumor volume (**E**) were measured on the indicated days. The arrows showed the time of doxorubicin injection. **F** FAM49B-shRNA or scr-shRNA were transfected into MDA-MB-231 cells treated with various doses of doxorubicin for 24 h. Levels of ELAVL1, Rab10, TLR4, p-p65, XIAP, survivin, c-caspase 3, and c-PARP1 in MDA-MB-231 cells were quantified via western blotting (n = 3). Results are presented as the mean ± SD. The statistical significance was assessed by Student’s *t*-test; *p < 0.05, **p < 0.01

ROC Plotter showed that the level of FAM49B mRNA in BC samples of anthracycline responders was significantly lower than that in BC samples of anthracycline non-responders (*p* = 5.3e-07, Fig. 9A) [25]. Furthermore, Kaplan–Meier survival analysis showed that high FAM49B mRNA expression was correlated with reduced RFS in BC patients who received chemotherapy (HR = 1.39, *p* = 0.019, Fig. 9B). These results suggest that high FAM49B expression may inhibit the chemosensitivity of BC. TNBC is generally malignant, and there are no effective targeted drugs. Therefore, chemotherapy is the main treatment method for TNBC [26]. To evaluate whether FAM49B can directly promote anthracycline resistance to chemotherapy in TNBC cells, MDA-MB-231-shFAM49B cells (or control MDA-MB-231 cells) were treated with doxorubicin. The levels of apoptosis were significantly higher in FAM49B-shRNA MDA-MB-231 cells than in control cells, following treatment with 200 ng/mL doxorubicin (*p* < 0.01, Fig. 9C). We used FAM49B-shRNA MDA-MB-231 cells (or control MDA-MB-231 cells) in a xenograft tumor model (Fig. 9D). The size of tumors formed by the control group cells was slightly reduced by doxorubicin treatment (*p* > 0.05, Fig. 9E), whereas the size of the tumors formed by FAM49B-shRNA cells was significantly reduced by doxorubicin treatment (*p* < 0.05, Fig. 9E). These results show that the expression of FAM49B is directly related to an increase in anthracycline resistance via inhibition of apoptosis.

Next, we determined whether FAM49B expression exerts anthracycline resistance in TNBC cells through ELAVL1 and the Rab10/TLR4 signaling pathway. After FAM49B knockout MDA-MB-231 cells were treated with doxorubicin, the protein levels of ELAVL1, Rab10, TLR4, phosphorylate-p65 (p-p65), XIAP, and survivin decreased in a dose-dependent manner (Fig. 9F). In addition, ELAVL1, Rab10, TLR4, p-p65, XIAP, and survivin protein levels were significantly lower in FAM49B-knockdown cells than in control cells following treatment with the corresponding doses of doxorubicin (*p* < 0.01, Fig. 9F). However, the protein levels of cleaved caspase 3 (c-caspase 3) and cleaved PARP1 (c-PARP1) were significantly higher in FAM49B-knockdown cells than in control cells following treatment with the corresponding doses of doxorubicin (*p* < 0.05, Fig. 9F). These results suggest that FAM49B may inhibit the apoptosis and pro-apoptotic protein activation in BC cells through the ELAVL1/Rab10/TLR4/NF-κB signaling pathway, resulting in anthracycline resistance.

## Discussion

To our knowledge, this is the first study to explore the role and mechanism of FAM49B in BC. ONCOMINE analysis revealed that FAM49B mRNA expression was significantly higher in BC samples than in normal samples, which corroborated with the observed elevation in its protein expression in BC samples, as assessed via western blotting and IHC, indicating that FAM49B has a potential impact on BC. Many scholars believe that BC is a heterogeneous disease because of its clinical and pathological characteristics and differences in molecular characteristics observed in multiple BC subtypes [27]. The prognosis of luminal type BC is often better than that of other subtypes because HER2-positive and TNBC subtypes are prone to recurrence and metastasis after treatment [27–29]. Our results revealed that FAM49B expression was negatively correlated with ER expression and positively correlated with HER2 expression. These results revealed that FAM49B expression correlated with the molecular subtype of BC, suggesting that BC with high expression of FAM49B is more malignant and aggressive.

Few studies have revealed that FAM49B is a potential tumor suppressor in pancreatic cancer, colorectal cancer, and liver cancer [9, 10]. Another study revealed that FAM49B promotes gallbladder carcinoma cell proliferation and migration [11], indicating that FAM49B is closely related to tumors. According to our results, high FAM49B mRNA expression is correlated with reduced OS and DFS in BC patients using Kaplan–Meier plotter survival analysis. Consistent with this, we found that the survival rate of patients positive for FAM49B expression was significantly lower than that of patients negative for FAM49B expression, in our BC samples. It shows that the expression of FAM49B is closely related to the survival time of breast cancer patients. In addition, our results revealed that FAM49B can significantly promote the proliferation and migration of BC cells. The above results indicate that FAM49B may play different roles in different types of cancer; and it has a clear potential as a new biomarker, providing a potential reference for the therapeutic effect of breast cancer. TLR4 is a member of the Toll-like protein family, mainly located in the cell membrane and cytoplasm, and it was initially studied in immune cells [30]. TLR4 protein expression strongly correlates with the expression of pro-inflammatory mediators and correlates with a decreased survival rate in patients with BC [31]. In addition, TLR4 plays an important role in increasing the efficiency of conventional anticancer treatments. Moreover, TLR4 has also been shown to promote metastasis in non-small cell lung cancer, hepatocellular carcinoma, oral squamous cell carcinoma, BC, and colon cancer [32–36]. According to our results, FAM49B knockdown significantly repressed the Toll-like receptor signaling pathway. In the toll-like receptor signaling pathway, when FAM49B is silenced, the expression of Rab10 and TLR4 mRNA is inhibited, indicating that FAM49B may act on BC by regulating Rab10 and TLR4, and TLR4 may be the downstream target of Rab10.

Rab10 is a protein coding gene with GTP- and GDP-binding domains and belongs to the RAS superfamily of small GTPases [37, 38]. A recent study demonstrated that Rab10 was highly expressed in liver cancer tissue samples [39, 40]. Furthermore, inhibition of Rab10 represses osteosarcoma cell proliferation and metastasis [41]. A previous study demonstrated that Rab10 regulated the transport of TLR4, which was vital for innate immune responses [42]. Our results show that Rab10 can inhibit the expression of TLR4 protein, and Rab10 is silenced when FAM49B is overexpressed, while the expression of TLR4 is significantly decreased, indicating that FAM49B can not only positively regulate the expression of Rab10 and TLR4, but also positively regulate the expression of Rab10 and TLR4. Moreover, Rab10 expression is essential for FAM49B to regulate BC cell pathway through TLR4. Rab10 has been studied as an oncogene in BC for the first time. However, based on the mechanism of regulation of Rab10 by FAM49B, ELAVL1 may be the downstream target of FAM49B and plays a central role in regulating Rab10, according to the IPA database.

**Fig. 10:**
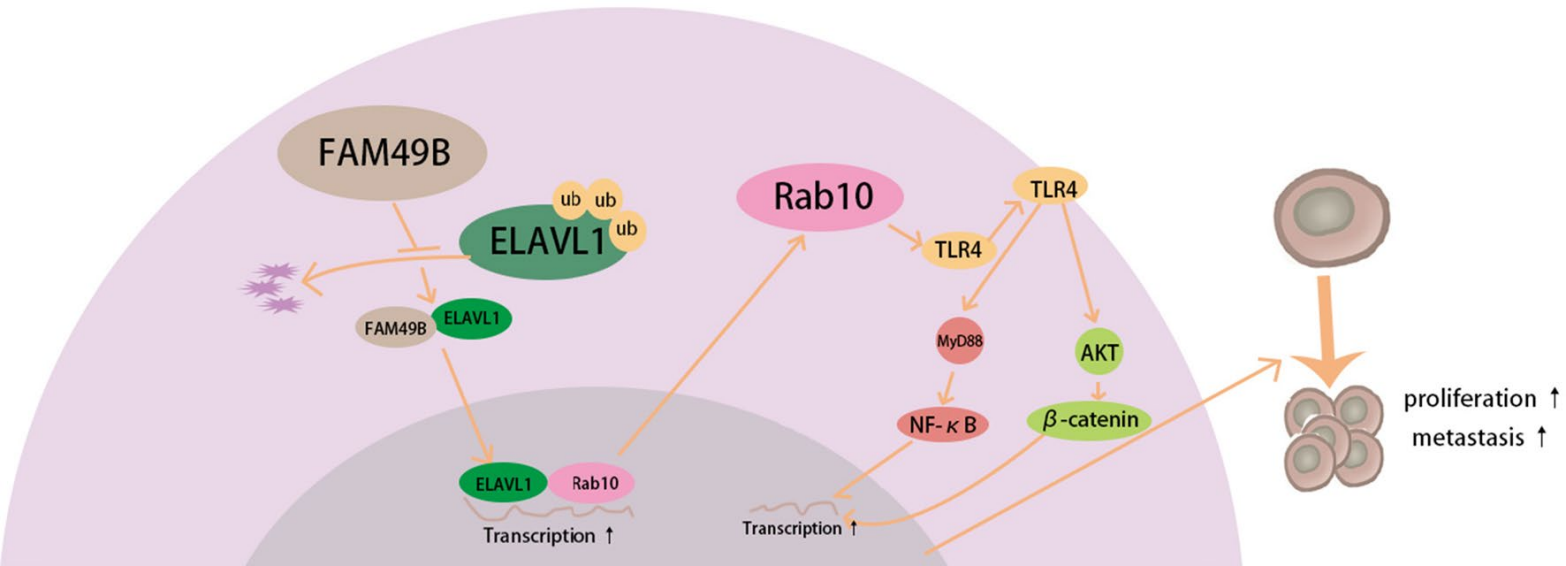
The signaling pathway proposed in this study

The RNA-binding protein ELAVL1 is one of the best-studied regulators of cytoplasmic mRNA fate [43]. Mechanistically, ELAVL1 regulates mRNA cargos that typically contain AREs in the 3ʹ-untranslated region, including numerous mRNAs involved in diverse biological processes of carcinogenesis [23, 44]. Elevated cytoplasmic ELAVL1 protein levels have been observed in many types of cancer [45]. The protein level of ELAVL1 was found to be modulated by the ubiquitin–proteasome pathway [46]. We analyzed the database to determine that ELAVL1 is a potential interaction factor of FAM49B. Our results show that endogenous FAM49B can interact with ELAVL1 in BC cells, and FAM49B knockdown does not change the expression of ELAVL1 mRNA, indicating that FAM49B stabilizes ELAVL1 expression at the post-translational level and prevents it from being ubiquitinated. In addition, our study found that silencing ELAVL1 during FAM49B overexpression significantly reduces the expression of Rab10 and TLR4, indicating that ELAVL1 positively regulates the expression of Rab10 and TLR4 in BC cells. Therefore, we speculate that FAM49B binds to ELAVL1 and stabilizes it to prevent its degradation via ubiquitination. ELAVL1 then increases the expression of Rab10 by binding to Rab10 mRNA. Later, Rab10 enhances the transport of TLR4 to the cell membrane and activates downstream pathways, such as NF-κB [47, 48] and Akt/GSK3β/β-catenin pathways [49], and ultimately promotes the proliferation and metastasis of BC cells (Fig. 10).

Chemotherapy is currently an effective treatment for TNBC. St Gallen experts recommend anthracyclines and taxanes as the main adjuvant chemotherapy drugs for TNBC. However, more than 50% of TNBC patients are resistant to adjuvant chemotherapy [50]. We confirmed through ROC Plotter online analysis that FAM49B mRNA levels in anthracycline responders in BC samples were significantly lower than non-responders in BC samples, indicating that FAM49B may be related to drug resistance, so we further studied its correlation. This study demonstrated that FAM49B knockout could significantly increase doxorubicin-induced apoptosis of cancer cells and the sensitivity of the TNBC xenograft tumor model to doxorubicin. Next, we further studied how FAM49B causes chemotherapy resistance. In BC cells, the increase in ELAVL1 in the cytoplasm is related to doxorubicin-induced apoptosis [51, 52]. Furthermore, chemotherapy may induce the expression of TLR4, leading to chemotherapy resistance. Therefore, inhibition of TLR4 with TAK-242 could reverse chemotherapy resistance [53, 54]. TLR4 activates the NF-κB pathway [55, 56] and downstream target genes XIAP and survivin [57]. XIAP and survivin directly inhibit the activity of caspase 3, which plays an important role in cell apoptosis and activation of poly ADP ribose polymerase (PARP) [58]. The results showed that FAM49B knockout in TNBC cells resulted in decreased ELAVL1, Rab10, TLR4, p-p65, XIAP, and survivin levels and increased c-caspase 3 and c-PARP1 levels. These results suggest that FAM49B knockdown can increase chemotherapy sensitivity, increase apoptosis and activation of apoptotic proteins, improve the effective rate of chemotherapy and improve the prognosis of breast cancer patients by down-regulating ELAVL1/Rab10/TLR4/NF-κB signaling pathway.

It is true that our research still has certain limitations. For example, the expression of FAM49B in five BC cells is not significantly different, which suppresses the potential of FAM49B as a biomarker for different molecular types of BC. In subsequent studies, we will continue to expand the sample size or try other methods to find specificity. The expression of FAM49B has been reported in other tumors, but its roles are different or even completely opposite. This suggests that FAM49B may have different roles in different developmental stages of cancer. In the future, we will continue to carry out the upstream and downstream targets of FAM49B. The research contributes to the research of FAM49B becoming a biomarker as soon as possible.

## Conclusion

In summary, we are the first to systematically study the mechanism of FAM49B mediated proliferation, metastasis and chemotherapy resistance of BC cells. This study showed that FAM49B can activate the proliferation and metastasis of BC cells through the ELAVL1/Rab10/TLR4 pathway, and the malignant degree and aggressiveness of breast cancer with high expression of FAM49B were significantly increased. FAM49B induces increased resistance of BC to anthracyclines through the ELAVL1/Rab10/TLR4/NF-κB pathway, and significantly reduces the chemotherapy effect and long-term survival rate of BC patients. Therefore, targeted suppression of FAM49B can not only reduce the malignancy of BC, but also greatly help patients with later chemotherapy and quality of life.

## Data Availability

Datasets supporting the conclusions of this article are included within the article.

## References

[1] Sung H, Ferlay J, Siegel RL, Laversanne M, Soerjomataram I, Jemal A, Bray F (2021). Global cancer statistics 2020: GLOBOCAN estimates of incidence and mortality worldwide for 36 cancers in 185 countries. CA Cancer J Clin.

[2] Shankar H, Saluja S, Rawat M, Singh G, Tarique M, Mohammad N, Naz H (2020). The causal association between occupational, environmental, and lifestyle factors and reproductive cancer risk. Curr Mol Biol Rep.

[3] Muhammad N, Steele R, Isbell TS, Philips N, Ray RB (2017). Bitter melon extract inhibits breast cancer growth in preclinical model by inducing autophagic cell death. Oncotarget.

[4] Federico C, Sun J, Muz B, Alhallak K, Cosper PF, Muhammad N, Jeske A, Hinger A, Markovina S, Grigsby P (2021). Localized delivery of cisplatin to cervical cancer improves its therapeutic efficacy and minimizes its side effect profile. Int J Radiat Oncol Biol Phys.

[5] Mohammad N, Singh SV, Malvi P, Chaube B, Athavale D, Vanuopadath M, Nair SS, Nair B, Bhat MK (2015). Strategy to enhance efficacy of doxorubicin in solid tumor cells by methyl-β-cyclodextrin: Involvement of p53 and Fas receptor ligand complex. Sci Rep.

[6] Prat A, Perou CM (2011). Deconstructing the molecular portraits of breast cancer. Mol Oncol.

[7] Nagarajan NA, Gonzalez F, Shastri N (2012). Nonclassical MHC class Ib-restricted cytotoxic T cells monitor antigen processing in the endoplasmic reticulum. Nat Immunol.

[8] Shang W, Jiang Y, Boettcher M, Ding K, Mollenauer M, Liu Z, Wen X, Liu C, Hao P, Zhao S (2018). Genome-wide CRISPR screen identifies FAM49B as a key regulator of actin dynamics and T cell activation. Proc Natl Acad Sci USA.

[9] Chattaragada MS, Riganti C, Sassoe M, Principe M, Santamorena MM, Roux C, Curcio C, Evangelista A, Allavena P, Salvia R (2018). FAM49B, a novel regulator of mitochondrial function and integrity that suppresses tumor metastasis. Oncogene.

[10] Long Y, Marian TA, Wei Z (2019). ZFR promotes cell proliferation and tumor development in colorectal and liver cancers. Biochem Biophys Res Commun.

[11] Zhang Y, Du P, Li Y, Zhu Q, Song X, Liu S, Hao J, Liu L, Liu F, Hu Y (2020). TASP1 promotes gallbladder cancer cell proliferation and metastasis by up-regulating FAM49B via PI3K/AKT pathway. Int J Biol Sci.

[12] Liu W, Zhang L, Jin Z, Zhao M, Li Z, Chen G, Sun L, Chen B (2017). TUFT1 is expressed in breast cancer and involved in cancer cell proliferation and survival. Oncotarget.

[13] Weiguang Liu JD, Zhao M, Zhang L, Jin Z, Chen B (2017). Clinical implications of TUFT1 protein expression and correlation with RelA protein in breast cancer. Int J Clin Exp Pathol.

[14] Chen G, Sun L, Han J, Shi S, Dai Y, Liu W (2019). RILPL2 regulates breast cancer proliferation, metastasis, and chemoresistance via the TUBB3/PTEN pathway. Am J Cancer Res.

[15] Ghebeh H, Al-Khaldi S, Olabi S, Al-Dhfyan A, Al-Mohanna F, Barnawi R, Tulbah A, Al-Tweigeri T, Ajarim D, Al-Alwan M (2014). Fascin is involved in the chemotherapeutic resistance of breast cancer cells predominantly via the PI3K/Akt pathway. Br J Cancer.

[16] Györffy B, Lanczky A, Eklund AC, Denkert C, Budczies J, Li Q, Szallasi Z (2010). An online survival analysis tool to rapidly assess the effect of 22,277 genes on breast cancer prognosis using microarray data of 1,809 patients. Breast Cancer Res Treat.

[17] Wang D, Lou J, Ouyang C, Chen W, Liu Y, Liu X, Cao X, Wang J, Lu L (2010). Ras-related protein Rab10 facilitates TLR4 signaling by promoting replenishment of TLR4 onto the plasma membrane. Proc Natl Acad Sci USA.

[18] Yue B, Song C, Yang L, Cui R, Cheng X, Zhang Z, Zhao G (2019). METTL3-mediated N6-methyladenosine modification is critical for epithelial-mesenchymal transition and metastasis of gastric cancer. Mol Cancer.

[19] Xue F, Li QR, Xu YH, Zhou HB (2019). MicroRNA-139-3p inhibits the growth and metastasis of ovarian cancer by inhibiting ELAVL1. Onco Targets Ther.

[20] Jain A, Agostini LC, McCarthy GA, Chand SN, Ramirez A, Nevler A, Cozzitorto J, Schultz CW, Lowder CY, Smith KM (2019). Poly (ADP) ribose glycohydrolase can be effectively targeted in pancreatic cancer. Can Res.

[21] Chen X, Li A, Sun BF, Yang Y, Han YN, Yuan X, Chen RX, Wei WS, Liu Y, Gao CC (2019). 5-methylcytosine promotes pathogenesis of bladder cancer through stabilizing mRNAs. Nat Cell Biol.

[22] Zhang Z, Yao Z, Wang L, Ding H, Shao J, Chen A, Zhang F, Zheng S (2018). Activation of ferritinophagy is required for the RNA-binding protein ELAVL1/HuR to regulate ferroptosis in hepatic stellate cells. Autophagy.

[23] Melling N, Taskin B, Hube-Magg C, Kluth M, Minner S, Koop C, Grob T, Graefen M, Heinzer H, Tsourlakis MC (2016). Cytoplasmic accumulation of ELAVL1 is an independent predictor of biochemical recurrence associated with genomic instability in prostate cancer. Prostate.

[24] Rouillard AD, Gundersen GW, Fernandez NF, Wang Z, Monteiro CD, McDermott MG, Ma'ayan A (2016). The harmonizome: a collection of processed datasets gathered to serve and mine knowledge about genes and proteins. Database (Oxford).

[25] Fekete JT, Győrffy B (2019). ROCplot.org: validating predictive biomarkers of chemotherapy/hormonal therapy/anti-HER2 therapy using transcriptomic data of 3104 breast cancer patients. Int J Cancer.

[26] Kast K, Link T, Friedrich K, Petzold A, Niedostatek A, Schoffer O, Werner C, Klug SJ, Werner A, Gatzweiler A (2015). Impact of breast cancer subtypes and patterns of metastasis on outcome. Breast Cancer Res Treat.

[27] Metzger-Filho O, Tutt A, de Azambuja E, Saini KS, Viale G, Loi S, Bradbury I, Bliss JM, Azim HA, Ellis P (2012). Dissecting the heterogeneity of triple-negative breast cancer. J Clin Oncol.

[28] Berry DA, Cirrincione C, Henderson IC, Citron ML, Budman DR, Goldstein LJ, Martino S, Perez EA, Muss HB, Norton L (2006). Estrogen-receptor status and outcomes of modern chemotherapy for patients with node-positive breast cancer. JAMA.

[29] Early Breast Cancer Trialists' Collaborative Group (2005). Effects of chemotherapy and hormonal therapy for early breast cancer on recurrence and 15-year survival: an overview of the randomised trials. Lancet (London, England).

[30] Haricharan S, Brown P (2015). TLR4 has a TP53-dependent dual role in regulating breast cancer cell growth. Proc Natl Acad Sci USA.

[31] Mehmeti M, Allaoui R, Bergenfelz C, Saal LH, Ethier SP, Johansson ME, Jirström K, Leandersson K (2015). Expression of functional toll like receptor 4 in estrogen receptor/progesterone receptor-negative breast cancer. Breast Cancer Res BCR.

[32] Liu X, Pei C, Yan S, Liu G, Liu G, Chen W, Cui Y, Liu Y (2015). NADPH oxidase 1-dependent ROS is crucial for TLR4 signaling to promote tumor metastasis of non-small cell lung cancer. Tumour Biol.

[33] Liu WT, Jing YY, Yu GF, Han ZP, Yu DD, Fan QM, Ye F, Li R, Gao L, Zhao QD (2015). Toll like receptor 4 facilitates invasion and migration as a cancer stem cell marker in hepatocellular carcinoma. Cancer Lett.

[34] He Z, Deng R, Huang X, Ni Y, Yang X, Wang Z, Hu Q (2015). Lipopolysaccharide enhances OSCC migration by promoting epithelial-mesenchymal transition. J Oral Pathol Med.

[35] Yang H, Wang B, Wang T, Xu L, He C, Wen H, Yan J, Su H, Zhu X (2014). Toll-like receptor 4 prompts human breast cancer cells invasiveness via lipopolysaccharide stimulation and is overexpressed in patients with lymph node metastasis. PLoS ONE.

[36] Santaolalla R, Sussman DA, Ruiz JR, Davies JM, Pastorini C, España CL, Sotolongo J, Burlingame O, Bejarano PA, Philip S (2013). TLR4 activates the β-catenin pathway to cause intestinal neoplasia. PLoS ONE.

[37] Vieira OV (2018). Rab3a and Rab10 are regulators of lysosome exocytosis and plasma membrane repair. Small GTPases.

[38] Isabella AJ, Horne-Badovinac S (2016). Rab10-mediated secretion synergizes with tissue movement to build a polarized basement membrane architecture for organ morphogenesis. Dev Cell.

[39] Zhang YJ, Pan Q, Yu Y, Zhong XP (2020). microRNA-519d induces autophagy and apoptosis of human hepatocellular carcinoma cells through activation of the AMPK signaling pathway via Rab10. Cancer Manag Res.

[40] Wang W, Jia WD, Hu B, Pan YY (2017). RAB10 overexpression promotes tumor growth and indicates poor prognosis of hepatocellular carcinoma. Oncotarget.

[41] Jiang W, Liu J, Xu T, Yu X (2016). MiR-329 suppresses osteosarcoma development by downregulating Rab10. FEBS Lett.

[42] Barbosa MD, Johnson SA, Achey K, Gutierrez MJ, Wakeland EK, Zerial M, Kingsmore SF (1995). The Rab protein family: genetic mapping of six Rab genes in the mouse. Genomics.

[43] Chang SH, Hla T (2014). Post-transcriptional gene regulation by HuR and microRNAs in angiogenesis. Curr Opin Hematol.

[44] Lan Y, Xiao X, He Z, Luo Y, Wu C, Li L, Song X (2018). Long noncoding RNA OCC-1 suppresses cell growth through destabilizing HuR protein in colorectal cancer. Nucleic Acids Res.

[45] Abdelmohsen K, Gorospe M (2010). Posttranscriptional regulation of cancer traits by HuR. Wiley Interdiscip Rev RNA.

[46] Abdelmohsen K, Srikantan S, Yang X, Lal A, Kim HH, Kuwano Y, Galban S, Becker KG, Kamara D, de Cabo R (2009). Ubiquitin-mediated proteolysis of HuR by heat shock. EMBO J.

[47] Piao W, Ru LW, Piepenbrink KH, Sundberg EJ, Vogel SN, Toshchakov VY (2013). Recruitment of TLR adapter TRIF to TLR4 signaling complex is mediated by the second helical region of TRIF TIR domain. Proc Natl Acad Sci USA.

[48] Wu X, Chen H, Wu M, Peng S, Zhang L (2020). Downregulation of miR-182-5p inhibits the proliferation and invasion of triple-negative breast cancer cells through regulating TLR4/NF-κB pathway activity by targeting FBXW7. Ann Transl Med.

[49] Li J, Yin J, Shen W, Gao R, Liu Y, Chen Y, Li X, Liu C, Xiang R, Luo N (2017). TLR4 promotes breast cancer metastasis via Akt/GSK3β/β-catenin pathway upon LPS stimulation. Anat Rec (Hoboken, NJ: 2007).

[50] Hong J, Chen XS, Wu JY, Huang O, Zhu L, He JR, Fang Q, Chen WG, Li YF, Shen KW (2017). Analysis of the factors influencing adjuvant chemotherapy decisions for triple negative breast cancer. Zhonghua zhong liu za zhi [Chin J Oncol].

[51] Mehta M, Basalingappa K, Griffith JN, Andrade D, Babu A, Amreddy N, Muralidharan R, Gorospe M, Herman T, Ding WQ (2016). HuR silencing elicits oxidative stress and DNA damage and sensitizes human triple-negative breast cancer cells to radiotherapy. Oncotarget.

[52] Latorre E, Tebaldi T, Viero G, Spartà AM, Quattrone A, Provenzani A (2012). Downregulation of HuR as a new mechanism of doxorubicin resistance in breast cancer cells. Mol Cancer.

[53] Kashani B, Zandi Z, Karimzadeh MR, Bashash D, Nasrollahzadeh A, Ghaffari SH (2019). Blockade of TLR4 using TAK-242 (resatorvid) enhances anti-cancer effects of chemotherapeutic agents: a novel synergistic approach for breast and ovarian cancers. Immunol Res.

[54] Zandi Z, Kashani B, Bashash D, Poursani EM, Mousavi SA, Chahardoli B, Ghaffari SH (2020). The anticancer effect of the TLR4 inhibition using TAK-242 (resatorvid) either as a single agent or in combination with chemotherapy: a novel therapeutic potential for breast cancer. J Cell Biochem.

[55] Lv W, Chen N, Lin Y, Ma H, Ruan Y, Li Z, Li X, Pan X, Tian X (2016). Macrophage migration inhibitory factor promotes breast cancer metastasis via activation of HMGB1/TLR4/NF kappa B axis. Cancer Lett.

[56] Sun T, Liu Y, Li M, Yu H, Piao H (2020). Administration with hyperoside sensitizes breast cancer cells to paclitaxel by blocking the TLR4 signaling. Mol Cell Probes.

[57] Tang G, Minemoto Y, Dibling B, Purcell NH, Li Z, Karin M, Lin A (2001). Inhibition of JNK activation through NF-kappaB target genes. Nature.

[58] Dan HC, Sun M, Kaneko S, Feldman RI, Nicosia SV, Wang HG, Tsang BK, Cheng JQ (2016). Akt phosphorylation and stabilization of X-linked inhibitor of apoptosis protein (XIAP). J Biol Chem.

